# Supplementary material for: Antibacterial Activity and Amphidinol Profiling of the Marine Dinoflagellate Amphidinium carterae (Subclade III)
Source: Int J Mol Sci. 2021 Nov 11;22(22):12196. doi: 10.3390/ijms222212196 (PMC8618426; doi:10.3390/ijms222212196)

Supplementary Material

Antibacterial Activity and Amphidinol Profiling of the Marine Dinoflagellate *Amphidinium carterae* (Subclade III)

Figure S1 HPLC-DAD-ELSD chromatograms of fraction I (210 nm).. 2

Figure S2 HPLC-DAD-ELSD chromatograms of fraction J (210 nm).. 2

Figure S3 EIC chromatogram of Amphidinol B at m/z 1464, BPC chromatogram of fraction G and MS/MS spectrum of Amphidinol B by C18 UHPLC-MS/MS.. 3

Figure S4 EIC chromatogram of Amphidinol 22 at m/z 1668, BPC chromatogram of fraction I by C18 UHPLC-MS/MS.. 4

Figure S5 EIC chromatogram of Amphidinol A at m/z 1362, BPC chromatogram of fraction I and MS/MS spectrum of Amphidinol A by C18 UHPLC-MS/MS.. 4

Figure S6 EIC chromatogram of Dehydroamphidinol A at m/z 1344, BPC chromatogram of fraction J and MS/MS spectrum of Dehydroamphidinol A by C18 UHPLC-MS/MS.. 4

Figure S1. HPLC-DAD-ELSD chromatograms of fraction I (210 nm).

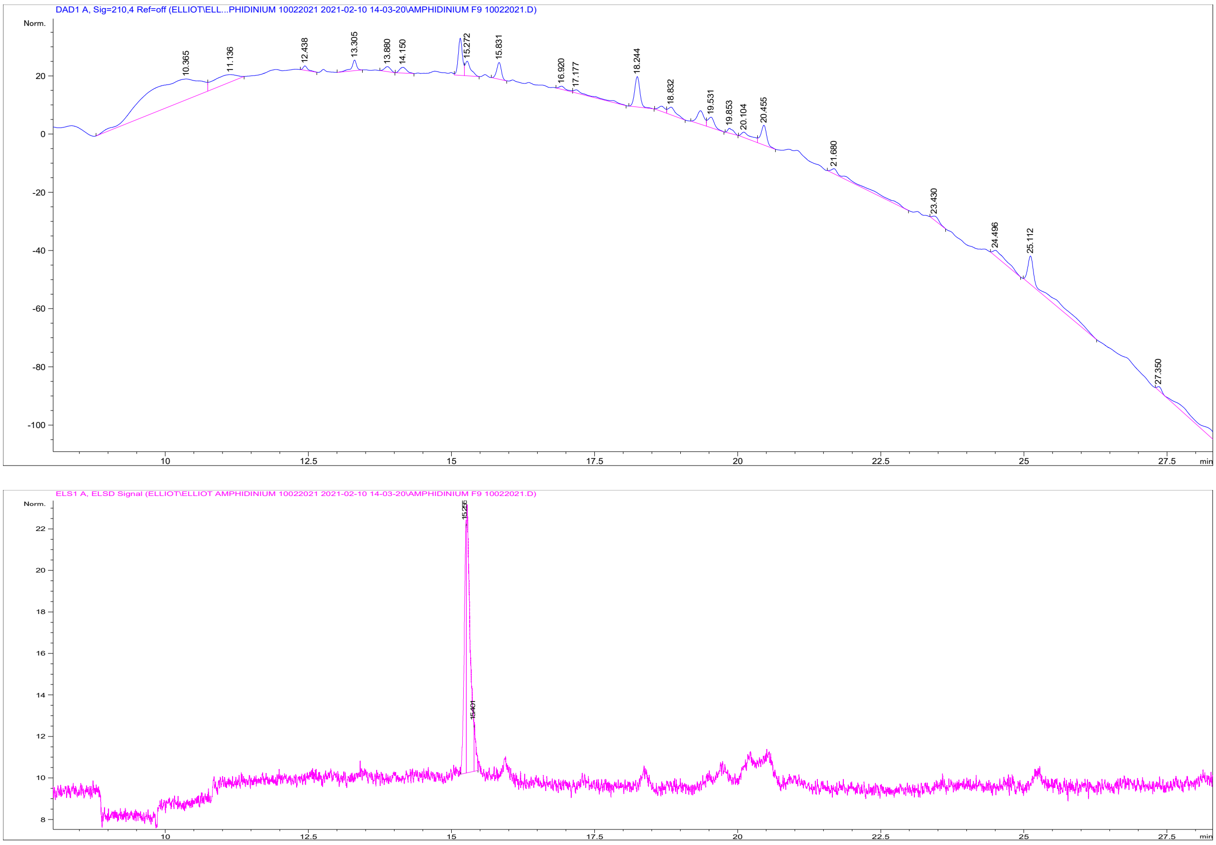

Figure S2. HPLC-DAD-ELSD chromatograms of fraction J (210 nm).

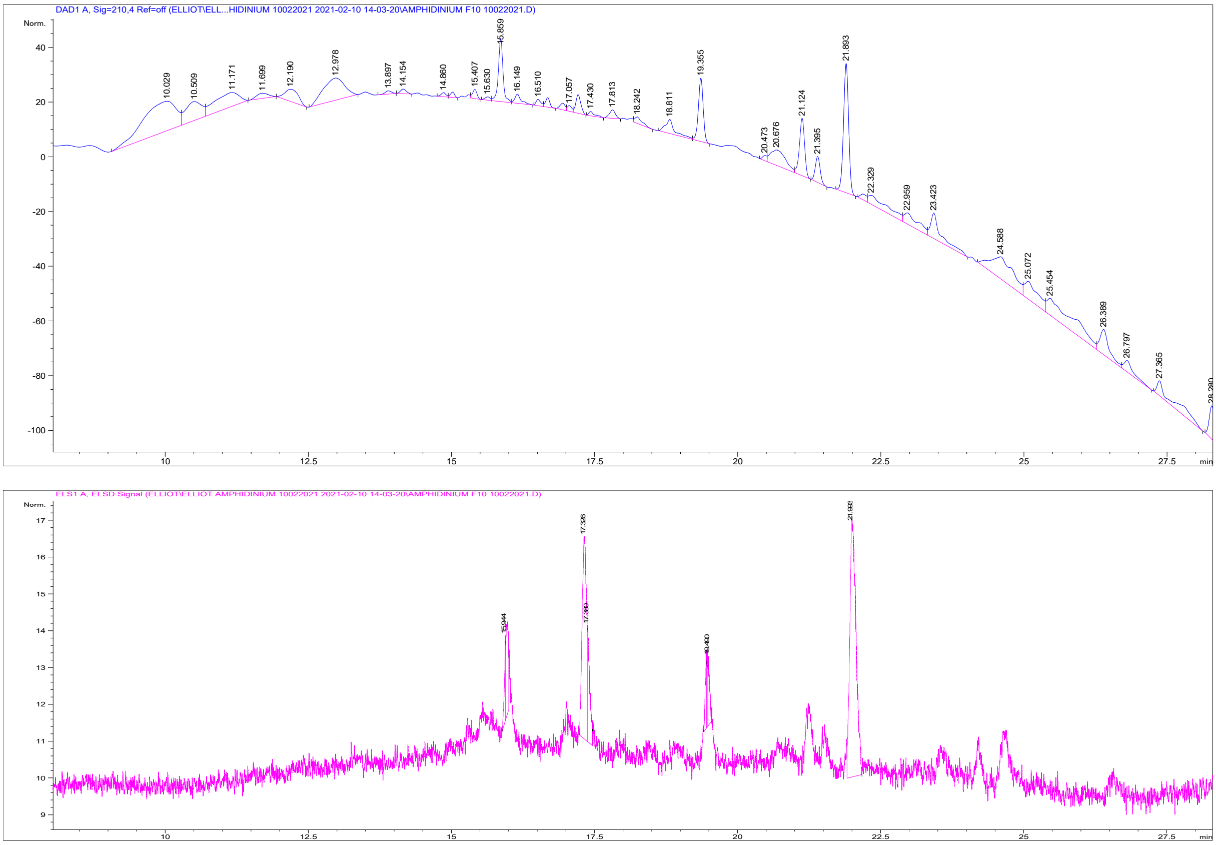

Figure S3. EIC chromatogram of Amphidinol B at *m/z* 1464, BPC chromatogram of fraction G and MS/MS spectrum of Amphidinol B by C18 UHPLC-MS/MS.

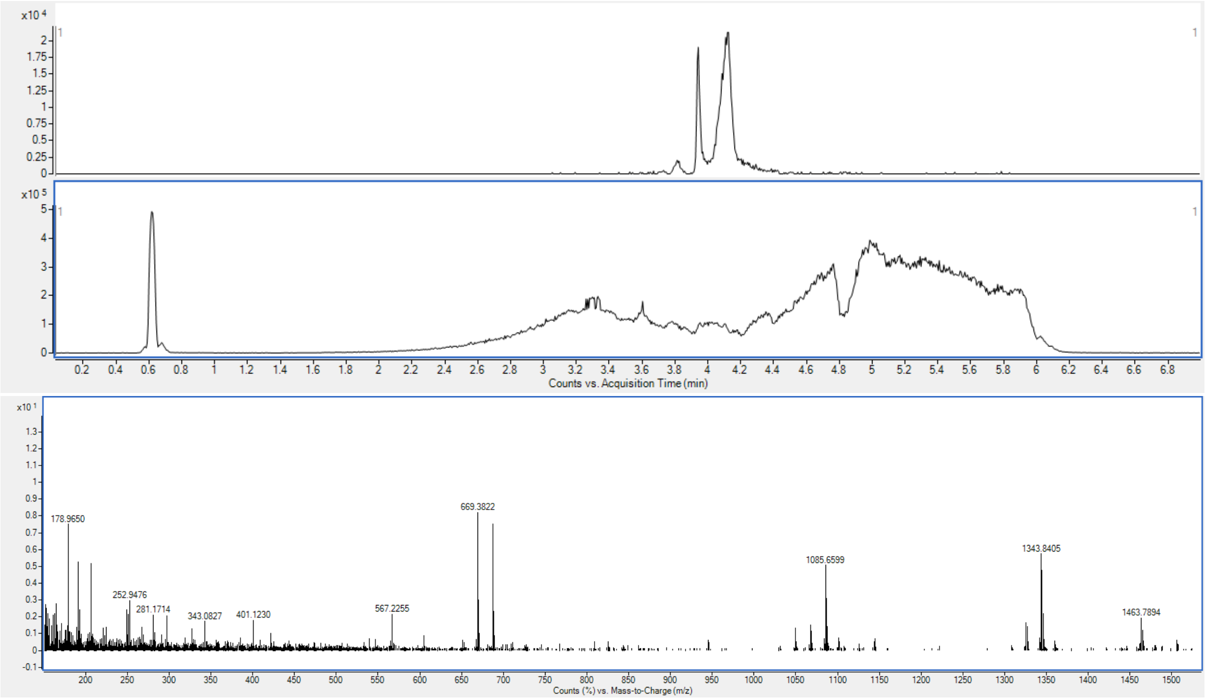

Figure S4. EIC chromatogram of Amphidinol 22 at *m/z* 1668, BPC chromatogram of fraction I by C18 UHPLC-MS/MS.

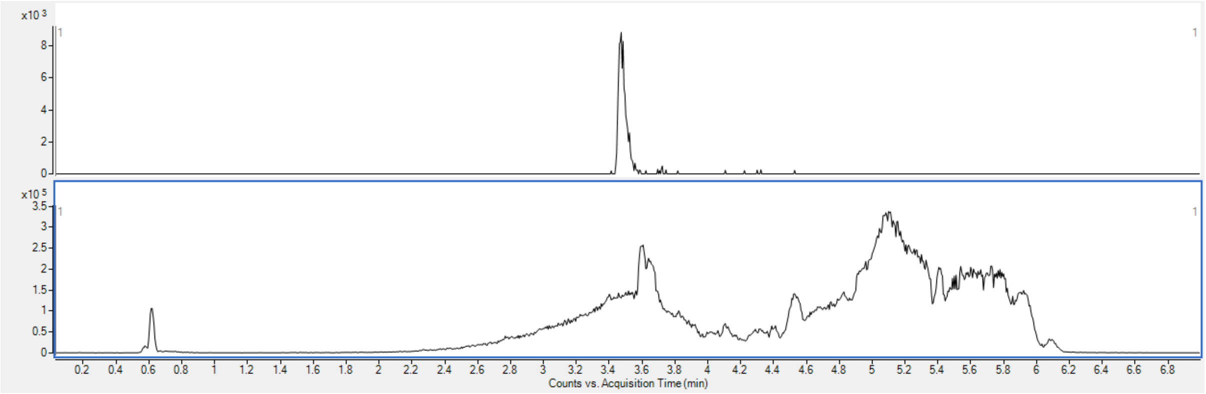

Figure S5. EIC chromatogram of Amphidinol A at *m/z* 1362, BPC chromatogram of fraction I and MS/MS spectrum of Amphidinol A by C18 UHPLC-MS/MS.

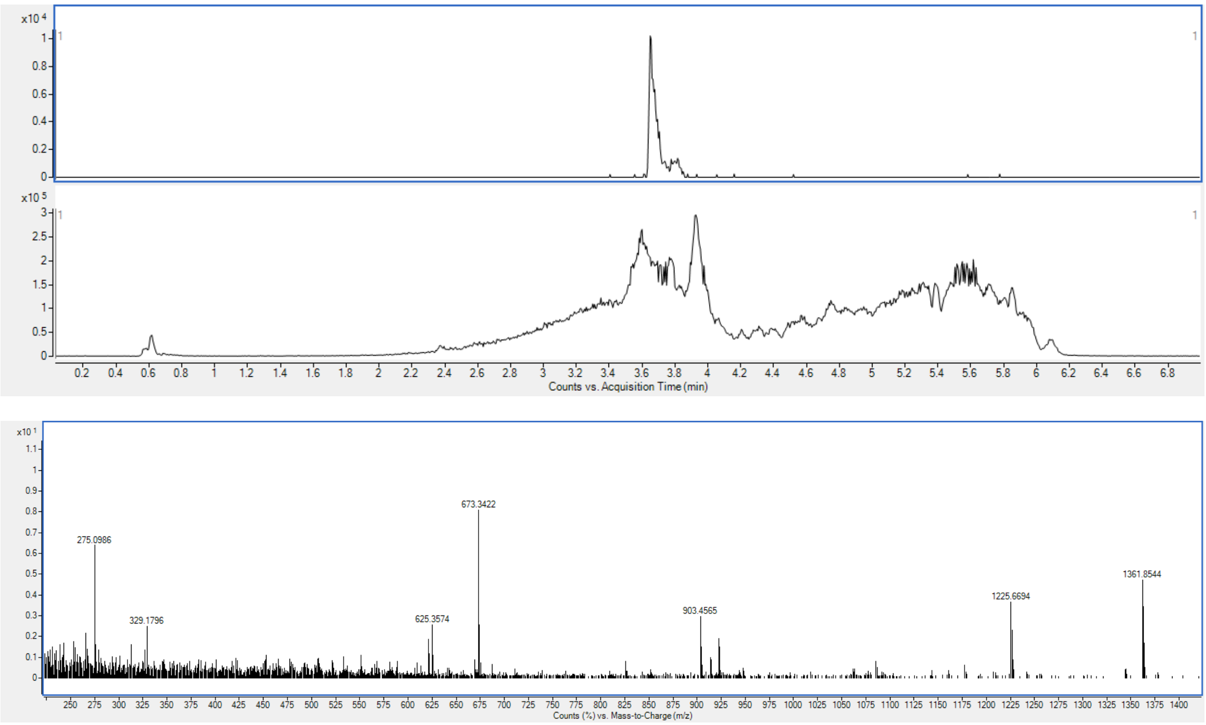

Figure S6. EIC chromatogram of Dehydroamphidinol A at *m/z* 1344, BPC chromatogram of fraction J and MS/MS spectrum of Dehydroamphidinol A by C18 UHPLC-MS/MS.

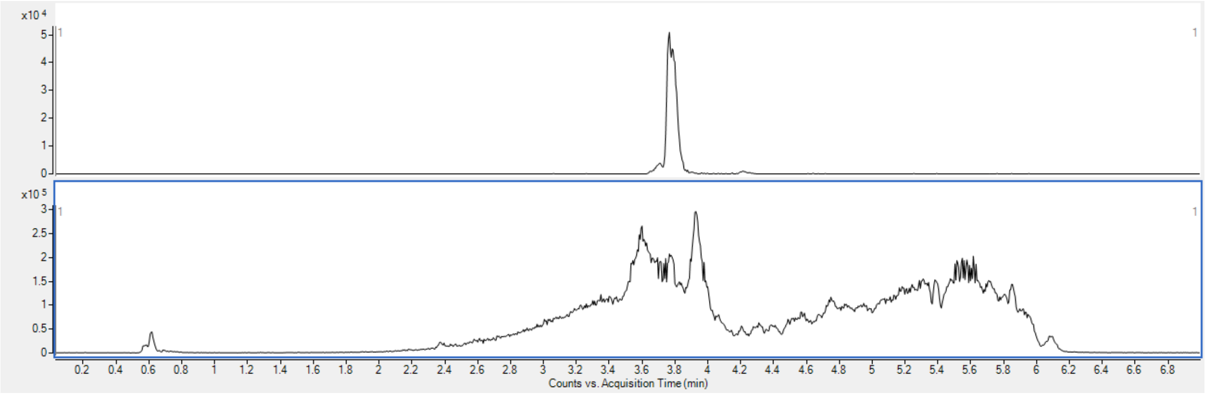

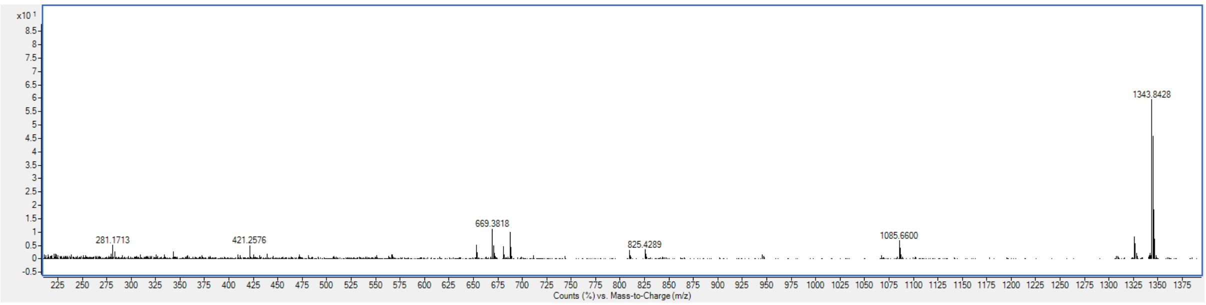

Supplement: Supplementary file 1 [file ijms-22-12196-s001.zip › ijms-1440142-supplementary.pdf]
